# Accessibility of long-term family planning methods: a comparison study between Output Based Approach (OBA) clients verses non-OBA clients in the voucher supported facilities in Kenya

**DOI:** 10.1186/s12913-017-2164-9

**Published:** 2017-03-27

**Authors:** Boniface Oyugi, Urbanus Kioko, Stephen Mbugua Kaboro, Shadrack Gikonyo, Clarice Okumu, Sarah Ogola-Munene, Shaminder Kalsi, Simon Thiani, Julius Korir, Paul Odundo, Billy Baltazaar, Moses Ranji, Nicholas Muraguri, Charles Nzioka

**Affiliations:** 1 University of Nairobi Enterprise and Services Consultancy, Arboretum Drive, P.O BOX 68241–00200, Nairobi, Kenya; 2 OBA Program Management Unit, Ministry of Health, Nairobi, Kenya; 3 Ministry of Health, Nairobi, Kenya

**Keywords:** Long term family planning, Output Based Approach, Voucher system

## Abstract

**Background:**

The study seeks to evaluate the difference in access of long-term family planning (LTFP) methods among the output based approach (OBA) and non-OBA clients within the OBA facility.

**Methods:**

The study utilises a quasi experimental design. A two tailed unpaired *t*-test with unequal variance is used to test for the significance variation in the mean access. The difference in difference (DiD) estimates of program effect on long term family planning methods is done to estimate the causal effect by exploiting the group level difference on two or more dimensions. The study also uses a linear regression model to evaluate the predictors of choice of long-term family planning methods. Data was analysed using SPSS version 17.

**Results:**

All the methods (Bilateral tubal ligation-BTL, Vasectomy, intrauterine contraceptive device -IUCD, Implants, and Total or combined long-term family planning methods -LTFP) showed a statistical significant difference in the mean utilization between OBA versus non-OBA clients. The difference in difference estimates reveal that the difference in access between OBA and non OBA clients can significantly be attributed to the implementation of the OBA program for intrauterine contraceptive device (*p* = 0.002), Implants (*p* = 0.004), and total or combined long-term family planning methods (*p* = 0.001). The county of residence is a significant determinant of access to all long-term family planning methods except vasectomy and the year of registration is a significant determinant of access especially for implants and total or combined long-term family planning methods. The management level and facility type does not play a role in determining the type of long-term family planning method preferred; however, non-governmental organisations (NGOs) as management level influences the choice of all methods (Bilateral tubal ligation, intrauterine contraceptive device, Implants, and combined methods) except vasectomy. The adjusted R^2^ value, representing the percentage of the variance explained by various models, is larger than 18% for implants and total or combined long-term family planning.

**Conclusion:**

The study showed that the voucher services in Kenya has been effective in providing long-term family planning services and improving access of care provided to women of reproductive age. Therefore, voucher scheme can be used as a tool for bridging the gap of unmet needs of family planning in Kenya and could potentially be more effective if rolled out to other counties.

## Background

Globally, there are more than 221 million women who wants to prevent unwanted pregnancies and assert their reproductive rights [1, 2]. Low and middle-income countries (LMIC) experience up to 99% of the 287,000 maternal deaths that happen globally per annum and family planning (FP) can prevent up to 30% of the deaths [1, 3]. In 2015, it was estimated that the contraceptives provided globally helped avert 34,000 maternal deaths, 4.4 million abortions (including 3.9 million unsafe ones), 12.3 million unintended pregnancies, and 220,000 child deaths [1].

Mother’s wellbeing as well as outcome of each pregnancy depends on the ability to limit and space her pregnancies. Consequently, increasing access to contraceptives can help alleviate poverty by improving public health outcomes [4]. Long term family planning (LTFP) methods such as intrauterine contraceptive devices (IUCDs), vasectomy, bilateral tubal ligation (BTL), and implants are current effective methods that prevents unwanted pregnancies [5]. LTFP are cost effective and when compared to short term methods such as injections and pills, result in fewer clinic visits and less unintended pregnancies; thereby, easing the burden on health systems and health providers [5, 6]. However, demographic health surveys from Sub-Saharan Africa (SSA) shows that most women are using short term methods than the long-term family planning methods [7]. Besides, the use of LTFP methods has declined over the past two decades with fewer than 5% of women of reproductive age in SSA using them [3, 8]. Several nations with poor resources have not yet achieved optimum levels of contraceptive and it is estimated that there is a 57% overall lack of contraceptive access within African countries [9].

There is still a gap in family planning usage that needs to be filled in Kenya since only 53% of currently married women aged 15–49 years and 61% of sexually active unmarried women are using contraceptives [10]. The unmet gap puts many women at a risk of unwanted pregnancies which could result in maternal deaths and unsafe abortions [2]. The unmet needs show a pressing health problem of access to contraceptive services which has pushed the donor and reproductive health community to prioritize family planning. Voucher programs such as output based approach (OBA) have been shown to enhance access to quality healthcare by the economically disadvantaged communities [11].

**Fig. 1:**
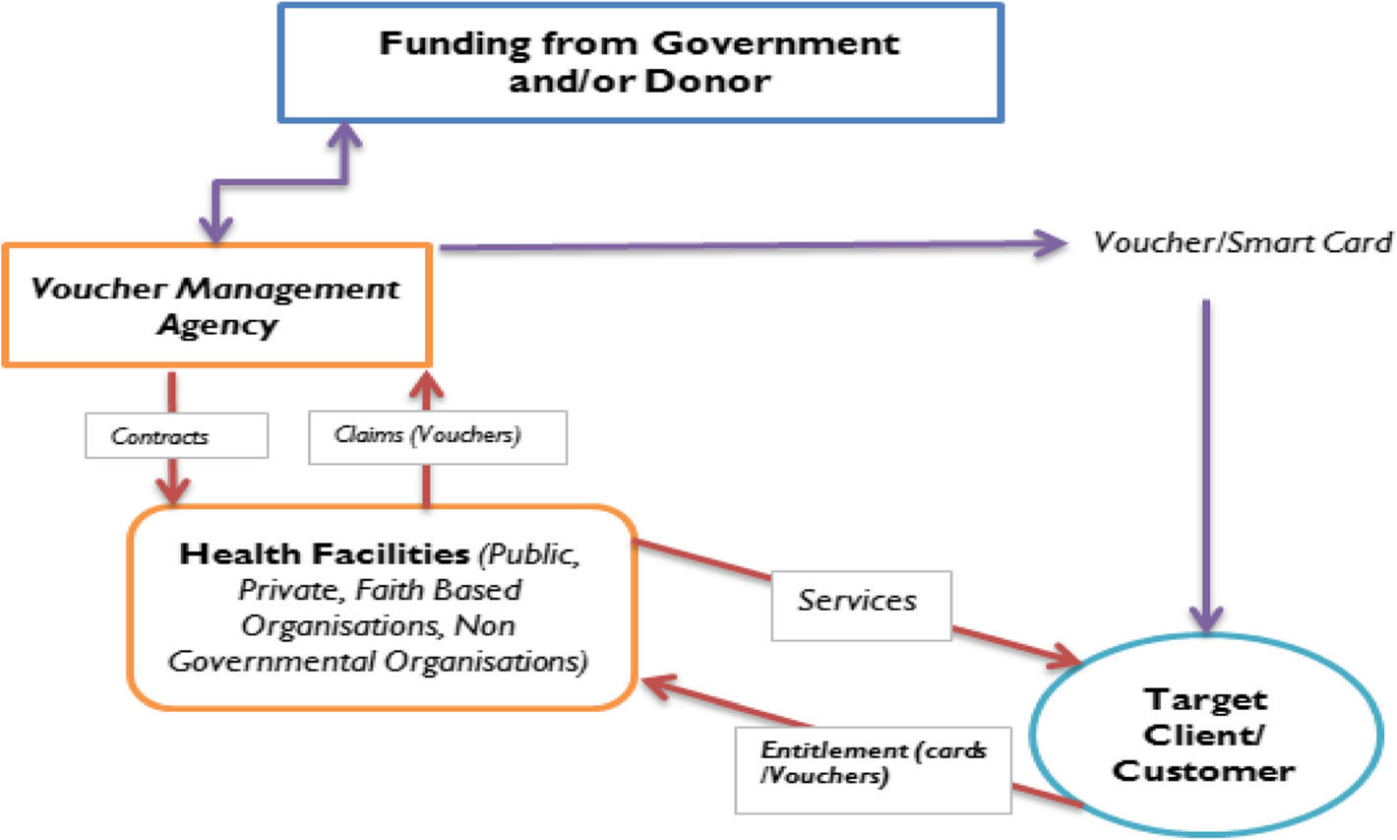
Basic Voucher Scheme

Voucher schemes were first used for family planning services between the 1960s and 1970s in Korea and Taiwan [12–14]. In voucher Schemes, subsidies are trickled-down from government and donors to underprivileged populations to stimulate demand for healthcare services. The coverage, design, and the services offered using the voucher schemes varies and are mainly determined by different governments’ areas of priority in collaboration with donors and financiers of the schemes [11]. Figure 1 shows a basic voucher scheme. The clients/consumers receive subsidies in voucher/smartcard form which are used in exchange for service from approved facilities (Public, faith based organizations, non-governmental organizations, and private) of choice. A number of providers are contracted in the scheme to enhance competition. The providers are contracted by a voucher management agency (VMA) normally competitively selected by the government or any other entity if implemented by a private agency [11].

Several studies have been done on the voucher management scheme in Kenya. For example; researchers have assessed the population level impact of vouchers on health facility delivery [15] and evaluated the community level impact of vouchers service utilization which showed that the voucher scheme helped in reducing the proportion of women in the community who paid out-of- pocket for safe motherhood services [16] Additionally, the quasi experimental evaluation of the voucher scheme showed the group level causal relationship between expansions of the Kenyan voucher mechanism and changes in the quality of post natal care [17]. On the other hand, other researcher have described the community experiences and perception of the clients on the voucher schemes [18] and a longitudinal analysis on facility based delivery in slums [19]. A comprehensive review worldwide voucher schemes revealed the lessons and practices learnt from different schemes [11] and a policy analysis on the reproductive voucher schemes described the implementation process of the Kenyan voucher program [20]. Of all the studies, there is none that evaluated the difference in access of LTFP methods using OBA clients compared to non- OBA clients within the same sites. Therefore this study seeks to evaluate the difference in access of LTFP methods among the OBA and non-OBA clients within the OBA facilities. We test the statistical significance of causal relationship between changes in access of LTFP methods and increasing the Kenya voucher program. Finally, we evaluate the predictors of choice of a specific method of LTFP besides social-demographic factors.

### Summary of Kenya’s voucher scheme

In Kenya, the voucher scheme herein referred to as Output Based Approach (OBA), was adopted with the support of the German Kreditanstalt für Wiederaufbau (KfW) Banking group. The scheme directly subsidizes access to high quality services including reproductive health (RH), increases the acceptance and utilization of family planning and gender based violence recovery services, and to reduce maternal and child mortality by the poor [21]. The OBA program was adopted as a flagship programme under the national Vision 2030 and sought to introduce social health insurance (SHI) and improve Public Private Partnerships (PPP) in attempt to widen the targeted group choice for service provider [21, 22]. The program was then designed in 2006 and has undergone three phases; Phase 1 from 2005–2008, Phase 2 from 2008–2011, which have been sufficiently described by other authors [20], and the third phase which started in November 2011 and is currently ongoing. The intervention is being implemented in Kenya’s Kitui (including Mwingi), Kilifi, Kiambu, and Kisumu (Including Nyando) counties as well as in the Korogocho and Viwandani slums in Nairobi. Mwingi and Nyando were added later in 2013. The health services in the chosen sites are run by public, NGO, FBO and private facilities as shown in Table 1 below. All the participating sites are offering safe motherhood and long term family planning methods and a small number are providing gender based violence recovery services free of charge at the facility to incentivize integrated service delivery, psychosocial support and legal services [19, 20, 23]. The clients who qualify to be holders of the voucher cards have to score sufficiently low on a 14-item scale including housing characteristics, water source and sanitation, existing access to healthcare, and income [19].

**Table 1:** The number of facilities in the OBA project

	FBO	NGO	Private	Public	totals
Kiambu	8	0	4	19	31
Nairobi	2	4	3	4	13
Kilifi	4	0	1	42	47
^a^Kisumu	13	1	9	38	61
^a^Kitui	7	0	5	64	76
Total	34	5	22	167	228

^a^Facilities in Nyando have been combined with the Kisumu, and Mwingi has been combined with Kitui

## Methods

### Study design, setting, and participants

The study utilises a quasi experimental design to evaluate the impact of OBA voucher scheme in Kenya on increasing access to LTFP methods by comparing number of OBA Clients who have accessed/used vouchers (herein referred to as the intervention group) and Non-OBA Clients who have not used vouchers (herein referred to as control group) but are in the same OBA facilities. Our study is based on the quantitative analysis of an existing data collected by the Voucher Management Agency (VMA) (National coordinating agency on population and development) of the OBA project in Kenya from 2008 to 2009, and existing data collected by the Voucher Management Agency (VMA) (Pricewaterhouse Coopers) from 2010 to 2015. Data collected prior to 2008 was not included in the analysis because it had no information on non-OBA clients (control group). In the OBA project, phase 1 comprise facilities that have been included since 2006 (program’s inception); phase 2 comprise facilities that started participating in the program from 2010; phase 3 comprise facilities that started participating since 2013. therefore, for this study data for 2008 was used as the baseline.

The study population consist of women of reproductive age (15–49 years) men both OBA and non-OBA clients who used LTFP methods (implants, IUCDs, tubal ligations, and vasectomy) in the 228 OBA accredited facilities (34 Faith Based Organisations, 5 Non-Governmental organisations, 22 private facilities, 167 public facilities) as shown in Table 1. A trained health provider from each of the accredited facilities (accreditation described elsewhere [24] recorded the number of clients both OBA (intervention group) and non-OBA (control group) who utilised any of the LTFP method in the OBA facilities (It is imperative to note that, the OBA sites also take care of non-OBA card holders who do not qualify for OBA cards). The records were done in a universal daily monitoring tool that was designed by the managing body (program management unit) that consisted of different sections for OBA and non-OBA client who had utilised LTFP methods. The data was nested in the longitudinal data set and then transmitted from the facilities to a central database at the VMA headquarters. The central database was monitored for errors by a VMA team member incharge of sytems management and the monitoring and evaluation officer incharge of data from the OBA program management unit of the Ministry of Health. This information was then stored in a computer based system at the program management unit (PMU) headquarters and updated on a monthly basis as part of the continuous monitoring process.

The location and the services covered by the OBA accredited facilities have been covered in the **summary of Kenya’s voucher scheme** above.

### Data analysis

In our study we compared the proportions OBA clients and non-OBA Clients who had used the LTFP methods in the OBA facilities since 2008 to 2015 and presented it in frequency and proportions. We tested for the significance variation in the mean number of OBA Clients who have accessed/used vouchers and non-OBA Clients who have not used vouchers but have accessed the LTFP methods within the same OBA facilities using two tailed unpaired *t*-test with unequal variance.

We further tested for the difference in difference (DiD) estimates of program effect on LTFP methods to estimate the causal effect by exploiting the group level difference on two or more dimensions. The diference in difference estimator is the average change in outcomes of treatment group (OBA Clients) by subtracting from the average change in the comparison group (non-OBA clients). The DiD analysis adjusted for the time invariant difference between the two groups and presents the results in DiD estimators. The DiD estimators of our study on the continous outcomes measures (numbers of LTFP methods utilised) were estimated using the multiple linear regression models. The DiD estimators are shown in two models: Model 1 included data sampling time (2008 vs 2009–2015) and treatment type (OBA clients vs non-OBA clients) and the DiD estimator, and Model 2 added the facility level, management level, and county of residence. The facility level in model two are defined by the level of the hospital whereby lowest level has clinics, nursing homes, and dispensary, second level health centres, third level has county and subcounty hospitals, and the last level has the referal hospitals. The management level are deternimed as Government (GoK)/public hospitals, Mission/Faith Based Organisations (FBOs), Non-Governmental Organisations (NGOs), and private facilities. The county of residence are Kenya’s Kitui (including Mwingi), Kilifi, Kiambu, and Kisumu (including Nyando) counties as well as in the Korogocho and Viwandani slums in Nairobi which were the implementation sites for the OBA program. The general DiD model is described as:

$$ y i t = {\beta}_0 + {\beta}_1{X}_t + {\beta}_2{T}_t + {\beta}_3{X}_i*{T}_t + {X}_i\gamma + {Z}_t\delta + {\varepsilon}_{i t} $$

where *T*_*t*_ is dummy for intervention time, *X*_*t*_ is dummy for facility level, *β*_*3*_ is DiD estimator, *X*_*i*_ is the dummy for management level, and *Z*_*t*_ is the dummy for county of residence *y*_*it*_ is the summative outcome scores examined as the dependent variable for individual *i* at facility *t.*

The third part of the analysis was a multi linear regression model which was used to study whether the facility level, management level, county of residence, month of registration, and year of registration by the OBA clients were predictors of access and use of LTFP vouchers as shown in Table 2.

**Table 2:** Definition and measurement of variables used in multi linear regression model

Variable definition	measurement
Outcome variable Access/use of LTFP method	BTL, Vasectomy, IUCD, Implants, Total LTFP methods (Continuous variables-numbers)
Independent variables	
Facility Level	1 = Level 2 (clinics nursing homes, and dispensary), 2 = level 3 (health centres), 3 = level 4(county and subcounty hospitals), 4 = level 5 (Referal hospitals)
Management Level	1 = GoK/Public, 2 = Mission/Faith-Based, 3 = Non-Governmental Organisation, 4 = Private
Month of registration	1 = January, 2 = February, 3 = March, 4 = April, 5 = May, 6 = June, 7 = July, 8 = August, 9 = September, 10 = October, 11 = November, 12 = December
Year of reg	1 = 2008, 2 = 2009, 3 = 2010, 4 = 2011, 5 = 2012, 6 = 2013, 7 = 2014, 8 = 2015
County of residence	1 = Kiambu, 2 = Kilifi, 3 = Kisumu, 4 = Kitui, 5 = Mwingi, 6 = Nairobi, 7 = Nyando

Data analysis was performed using SPSS version 17.

### Ethical approval

The authorization to carry out the study was obtained from the Ministry of Health-Kenya as part of routine monitoring of the process (Development of the Health Sector, Health Financing Support and Output Based Approach, Phase III, BMZ-No. KENYA 2010 65853) of the OBA services. The proposal was approved by the health research unit of the Ministry of Health Kenya (MOH/HRD/1/[25]). Additionally, permission was obtained from the county headquarters and hospital administrators to proceed with the study. Further ethical approval was not necessary as the analysis and review relies on routine data collected by VMA.

## Results

### Use of long term family planning method

The number of OBA clients who have utilized LTFP methods is two times more than the Non-OBA clients. Implants are the most preferred long-term method by both OBA clients (89.6%) and non-OBA clients (72.4%) while IUCD is the second most preferred method by both OBA (8.6%) and Non-OBA clients (21.2%). Vasectomy is the least chosen method amongst OBA and non OBA clients as shown in Table 3.

**Table 3:** Descriptive characteristics of the OBA clients versus Non-OBA clients

	OBA Clients	Non OBA Clients
	Frequency (%)	Frequency (%)
BTL	4,568 (3.3)	3,771 (5.6)
Vasectomy	226 (0.16)	150 (0.22)
IUCD	12,031 (8.6)	14,341 (21.2)
Implants	125,440 (89.6)	48,873 (72.4)
Totals LTFP	139,946 (67.6)	67,514 (32.5)

Note: percentages may not sum to exactly 100 in some cases due to rounding percentages for the total LTFP is calculated by taking of either OBA or Non-OBA clients divided by the sum of OBA and non-OBA clients times 100%

### Difference in utilization level among users of OBA facilities

The difference in the mean utilization level of the clients involved in the study was checked with respect to the method of choice of LTFP method. Based on the independent sample *t* test performed on the method of choice, all the methods (BTL, Vasectomy, IUCD, Implants, Total LTFP) showed a statistical significant difference as shown in Table 4. The OBA clients who had chosen BTL, Vasectomy, Implants, and total use, were shown to have a higher level of mean utilization as compared to Non-OBA clients. On the other hand, the Non-OBA clients who had chosen IUCDs were shown to have a higher level of mean utilization as compared to OBA clients Table 4. Overall, the total utilization of LTFP method among OBA clients is significantly higher compared to non-OBA clients.

**Table 4:** Test of significance (Independent samples *t*-test) variation in the mean number of OBA Clients who have accessed/used vouchers and Non-OBA Clients who have not used vouchers but are in the same OBA facilities

Method		Mean (S.E)	SD	*p*-value
BTL	OBA	0.44 (0.036)	3.7	0.001*
	Non OBA	0.36 (0.24)	2.5	
Vasectomy	OBA	0.02 (0.005)	0.5	0.013*
	Non OBA	0.01(0.003)	0.3	
IUCD	OBA	1.15 (0.041)	4.2	<0.001*
	Non OBA	1.37 (0.46)	4.7	
Implants	OBA	11.98 (0.374)	38.3	<0.001*
	Non OBA	4.67 (0.14)	14.3	
Total LTFP	OBA	13.37 (0.384)	39.2	<0.001*
	Non OBA	6.45 (0.19)	19.1	

*p- value <0.05

### Difference in difference estimates of program effect on LTFP methods

Access was evaluated on all the categories of LTFP methods (BTL, Vasectomy, IUCD, Implants, and Total LTFP). The difference in difference estimates reveal that the difference in access between OBA and non-OBA clients can significantly be attributed to the implementation of the OBA program for IUCD (p = 0.002), Implants (p = 0.004), and total LTFPM methods (p = 0.001) as shown in Table 5 model 2. The B values have been given direct interpretation for instance; a 1.316 unit decrease in access of IUCD by Non-OBA clients is equivalent to a 1 unit increase in access by OBA clients controlling for facility type, management type, and residence. A 7.981 unit decrease in access of implants by Non-OBA clients is equivalent to a 1 unit increase in access by OBA clients in the preferred model 2. However, the difference in difference estimates shows that the difference in access between OBA and non OBA clients cannot be significantly attributed to the implementation of the OBA program for BTL (p = 0.366) and Vasectomy (p = 0.490).

**Table 5:** Difference in difference estimates of program effect on LTFP methods

Method	Difference in difference estimates		Difference in difference estimates	
	Model 1		Model 2	
	B (SE)	*p*-value	B (SE)	*p*-value
BTL	−0.107 (0.30)	0.726	−0.271 (0.30)	0.366
Vasectomy	−0.018 (0.04)	0.660	−0.028 (0.04)	0.490
IUCD	**−1.181 (0.43)**	0.006*	**−1.316 (0.43)**	0.002*
Implants	**−7.459 (2.84)**	0.009*	**−7.981 (2.75)**	0.004*
Total LTFP	**−8.670 (3.02)**	0.004*	**−9.494 (2.93)**	0.001*

*p- value <0.05

There is small variation in the estimated coefficients on the access scores for the LTFP methods (BTL, Vasectomy, IUCD, Implants, Overall/Total LTFP) between the crude model 1 and preferred model 2 which includes facility type, management type, and county of residence as shown in Table 5. Therefore facility type, management type, and county of residence do not influence the access by OBA or Non-OBA clients.

### Subgroup analysis

The associations were studied using the multivariate response models which allow the simultaneous inclusion of various dependent variables in the regression analysis, and improve quality of estimators. The B value was given a direct interpretation as shown in Table 6 and Table 7. Our study shows that the level of the facility and month of registration does not show significance in accessing any LTFP method which is inconsistent with the notes that the voucher management agency (VMA) has been including in the quarterly reports. The county of residence is a significant determinant of access to all LTFP method except vasectomy; however, a value 0.88 for vasectomy means that the OBA card holders in Kilifi have a 0.88 higher access to vasectomy than any other county, after adjusting for facility level, management level, month of registration, year of registration and county of residence. The year of registration is a significant determinant of access especially for implants and combined LTFP methods. The management level and facility type does not play a role in determining the type of LTFP method preferred; however, NGOs as management level influences the choice of all methods (BTL, IUCD, Implants, combined methods) except vasectomy as shown in Table 6 and Table 7.

**Table 6:** Methods of long term family planning (BTL, Vasectomy, IUCD) related access: multivariate response model

Independent Variable		Dependent Variable								
		BTL			Vasectomy			IUCD		
		B^a^	LB	UB	B	LB	UB	B	LB	UB
	Intercept	.948	−2.212	4.107	-.035	-.510	.440	1.650	−1.916	5.216
Facility Level	(Ref: 2)									
	3	-.028	−3.168	3.111	-.013	-.486	.459	−1.111	−4.656	2.433
	4	.043	−3.097	3.184	.059	-.413	.532	-.998	−4.543	2.547
	5	1.757	−1.404	4.919	-.017	-.492	.459	−1.222	−4.791	2.347
Management Level	(Ref: Gok1)									
	Mission2	.182	−2.949	3.314	.015	-.456	.486	.667	−2.868	4.201
	NGO3	**4.468** ^**b**^	1.320	7.617	.096	-.378	.570	3.275*	-.280	6.829
	Private	.657	−2.472	3.785	.010	-.461	.480	1.616	−1.916	5.147
Month of registration	(Ref: Jan)									
	Feb	-.808	−3.948	2.332	.003	-.469	.476	1.287	−2.258	4.831
	March	−1.041	−4.181	2.099	.011	-.462	.483	1.162	−2.383	4.707
	April	−1.091	−4.232	2.050	.005	-.467	.478	1.286	−2.259	4.832
	June	−1.016	−4.155	2.124	.028	-.444	.500	1.447	−2.097	4.991
	June	-.888	−4.027	2.250	.021	-.452	.493	1.273	−2.270	4.816
	July	-.934	−4.073	2.205	.006	-.467	.478	1.377	−2.166	4.921
	Aug	-.656	−3.795	2.482	.059	-.413	.531	1.326	−2.216	4.869
	Sept	-.856	−3.995	2.282	.019	-.453	.491	1.573	−1.970	5.115
	Oct	-.972	−4.110	2.166	.009	-.463	.481	1.443	−2.100	4.985
	Nov	-.993	−4.132	2.147	.007	-.465	.480	1.478	−2.066	5.022
	Dec	−1.125	−4.265	2.015	.006	-.466	.479	.864	−2.681	4.409
Year of reg	(Ref: 2014)									
	2008	.115	-.604	.835	-.021	-.129	.087	**−1.358**	−2.170	-.546
	2009	.281	-.046	.609	-.029	-.078	.020	**−1.228**	−1.598	-.859
	2010	**.470**	.164	.777	-.019	-.066	.027	-.187	-.534	.159
	2011	**.669**	.410	.927	-.034	-.072	.005	.196	-.096	.488
	2012	**.772**	.510	1.033	-.020	-.059	.019	.225	-.070	.521
	2013	.033	-.184	.249	-.016	-.048	.017	-.053	-.297	.192
	2015	-.015	-.223	.193	.030	-.001	.061	.120	-.115	.355
County of residence	(Ref: Kiambu)									
	Kilifi	**-.183**	-.421	0.56	**.088**	.052	.124	**−2.889**	−3.158	−2.619
	Kisumu	**-.335**	-.555	-.115	.003	-.030	.036	**−2.789**	−3.038	−2.540
	Kitui	**.276**	.055	.497	.026	-.008	.059	**−2.986**	−3.235	−2.736
	Mwingi	-.004	-.314	.306	.009	-.038	.055	**−2.907**	−3.257	−2.558
	Nairobi	**−1.511**	−1.815	−1.208	.002	-.044	.048	**−2.115**	−2.457	−1.772
	Nyando	-.173	-.473	.127	.002	-.043	.047	**−2.179**	−2.518	−1.841
Variance explained (R^2^)		6.3%			0.9%			8.8%		

a. The B values shown are interpreted directly: for instance, 4.468 for NGO on BTL means that for every 4.468 unit increase in access of BTL by individual in NGO there is a 1 unit increase in access of BTL by individuals in GOK, after adjusting for other variables such as facility level, Month of registration, year of registration, and county of residence

b. The bold values are significant at *p* < 0.05 while the bold values that have an asterisk (*) mark are significant at *p* = 0.1

**Table 7:** Methods of long term family planning (Implants and combined LTFP) related access: multivariate response model

Independent Variable		Dependent Variable					
		Implants			Combined LTFP		
		B^c^	LB	UB	B	LB	UB
	Intercept	25.735*	−4.833	56.303	28.078*	−2.985	59.140
Facility Level	(Ref: 2)						
	3	−10.401	−40.780	19.978	−11.734	−42.605	19.137
	4	−16.139	−46.525	14.247	−17.042	−47.919	13.836
	5	−14.566	−45.153	16.021	−14.651	−45.733	16.431
Management Level	(Ref: Gok1)						
	Mission2	6.244	−24.053	36.540	7.031	−23.756	37.817
	NGO3	**91.769**	61.304	122.233	**99.038**	68.080	129.995
	Private	14.524	−15.744	44.791	16.921	−13.837	47.678
Month of registration	(Ref: Jan)						
	Feb	−11.386	−41.767	18.995	−10.760	−41.633	20.113
	March	−11.454	−41.838	18.930	−10.896	−41.772	19.979
	April	−10.686	−41.075	19.702	−10.059	−40.939	20.822
	June	−10.007	−40.381	20.367	−9.245	−40.111	21.621
	June	−11.498	−41.867	18.871	−10.762	−41.623	20.098
	July	−10.603	−40.975	19.770	−9.787	−40.651	21.077
	Aug	−10.865	−41.231	19.501	−10.071	−40.929	20.786
	Sept	−9.691	−40.056	20.673	−9.314	−40.170	21.542
	Oct	−9.386	−39.750	20.978	−8.609	−39.464	22.247
	Nov	−10.103	−40.480	20.274	−9.410	−40.278	21.459
	Dec	−15.297	−45.682	15.088	−15.270	−46.146	15.607
Year of reg	(Ref: 2014)						
	2008	**−14.287**	−21.248	−7.325	**−15.343**	−22.417	−8.269
	2009	**−15.489**	−18.658	−12.319	**−16.212**	−19.433	−12.991
	2010	**−4.620**	−7.587	−1.652	**−4.135**	−7.151	−1.119
	2011	1.468	−1.032	3.969	2.259*	-.283	4.800
	2012	**5.904**	3.370	8.438	**6.424**	3.849	8.999
	2013	.161	−1.934	2.257	-.213	−2.342	1.917
	2015	−1.959*	−3.972	.054	−1.815*	−3.860	.231
County of residence	(Ref: Kilifi)						
		**−9.307**	−11.617	−6.997	**−12.214**	−14.562	−9.867
	Kisumu	**7.417**	5.287	9.548	**4.070**	1.905	6.235
	Kitui	**−8.072**	−10.209	−5.934	**−10.651**	−12.823	−8.479
	Mwingi	**−10.493**	−13.488	−7.498	**−13.014**	−16.057	−9.970
	Nairobi	**−15.212**	−18.147	−12.277	**−18.923**	−21.906	−15.941
	Nyando	−3.391*	−6.294	-.487	**−5.576**	−8.527	−2.625
Variance explained (R^2^)		19.7%			20.9%		

c. The B values shown are interpreted directly: for instance, 4.468 for NGO on BTL means that for every 91.769 unit increase in access of BTL by individual in NGO there is a 1 unit increase in access of BTL by individuals in GOK, after adjusting for other variables such as facility level, Month of registration, year of registration, and county of residence

d. The bold values are significant at *p* < 0.05 while the bold values that have an asterisk (*) mark are significant at *p* = 0.1

The adjusted R^2^ value, representing the percentage of the variance explained by various models, is larger than 18% for implants and combined LTFP. NGOs, year of registration and county of residence explain much of the variance. This shows that in our model, there is a high level of access of implants and combined LTFP methods relative to BTL, vasectomy and IUCDs.

## Discussion

One of the key goals of the voucher management system is to increase access and utilization of long term family planning (LTFP) methods [11, 20, 23, 26–29] and; thus, this paper evaluated access of LTFP methods by the women of reproductive age in the voucher scheme or OBA program in Kenya. One major finding is that there are two time more OBA clients who have utilized LTFP methods as compared to non-OBA clients within the OBA facilities. The increase in access among the OBA clients could be attributed to an increased efforts of the target community opinion leaders advocating for LAFP acceptance within the counties and the marketing strategies that had been developed by the VMA agencies across the years [30, 31]. While this study did not evaluate the trends and the difference in access within individual counties, there may have been a difference in access per county when it comes to using LTFP and; thus, future research can focus on highlighting the difference in counties.

The other finding of our study is that implants are the most preferred LTFP method by both OBA clients and non-OBA clients while IUCD is the second most preferred method. Vasectomy is the least chosen method amongst OBA and non OBA clients. The finding concurs with a review done in SSA which showed that implant was the preferred method of family planning because it protects against ovarian cancer, decreases a mother’s risk of anaemia, and pain and cramps associated with menstruation while IUCD lowers a woman’s risk of endometrial cancer [5]. While our study showed that vasectomy was the least preferred by the clients, the study on SSA revealed that there was no medical condition that would restrict an individual’s eligibility for vasectomy [5]. Although the study showed that implants were preferred, OBA quarterly reports revealed that in some instances some women insisted on getting consent from their husband before taking up the family planning services and some requested the nurse in-charge to only perform the implant insertions in different parts of the body other than the normal location as stated in the family planning guideline hence affecting the access [30, 31].

Our study further showed a statistical significant difference in access of LTFP methods between OBA verses non-OBA Clients. There was a higher utilization among OBA clients who had chosen BTL, vasectomy, and implants as compared to non-OBA clients while the non-OBA clients who had chosen IUCDs were shown to have a higher level of mean utilization as compared to OBA clients. Overall, the total utilization of LTFP method among OBA clients is significantly higher compared to Non OBA clients. The findings are supported by a study done in Kenya which showed that increased utilization amongst OBA clients may have been due to increased family planning advice by the health care providers in the OBA facilities [17]. Since vouchers schemes subsidizes on LTFP methods at voucher facility, it is expected that there is an increased supply of the methods within the facility and thus healthcare providers providing such services to OBA clients. The results are congruent to a study done in Kenya which showed that there may have been an increased utilization in family planning and thus spillover effect as a result of stocking contraceptive methods within the facilities [17].

The study showed that increased access of total or combined LTFP methods, IUCD, and implants is attributed to voucher scheme as evidenced by the difference in difference estimates. The finding is inconsistent with other literature which showed that ever use of LTFP methods might not be attributed to the program [16]. Although our study does not show that access of BTL and vasectomy can be attributed to voucher scheme, it still reveal that more OBA clients than non-OBA clients are using the two methods. Nevertheless, the finding is consistent with other literature that shows that demand side financing improves uptake of services [25, 26, 32]. The results could be attributed to the fact that the voucher service distributers (VSDs) have also been used to cover areas that were considered long distance from the facilities which aided in eliminating the obstacles to utilizing the methods in addition to explaining the benefits of long acting and permanent family planning methods [31]. The other plausible cause is that some mothers prefer to be OBA clients for contraceptives because some hospitals are now giving first priority to long acting family planning clients, so that they do not have to queue at the maternal and child health/family planning clinic which is a motivating factor thus increased access. In Nicaragua, voucher holders were equally given preferential treatment at the waiting rooms [25]. Therefore, output based approach scheme can be effective in bridging the unmet needs of family planning in Kenya and thus, may increase access in the hard to reach areas if rolled out to other counties.

Finally, our study showed that the county of residence, year of registration in the voucher scheme, and NGOs as management level influences the choice of LTFP methods. On the other hand, the level of the facility, and month of registration, does not influence choice of any LTFP methods which is a new finding added to existing literature. The results are consistent with other findings in the literature which showed place of residence played a role in uptake of voucher services for which LTFP is included [16]. The finding on NGO influencing the choice of LTFP could be attributed to the fact that in the spirit of competition within the voucher management system, private facilities and the non-profit organizations have been going a further mile in incentivizing the women by using their own revenue to purchase the OBA vouchers/Smart cards for women to enable them access the services. However, this has a potential of raising issues with quality skimping especially when the facility have taken greater number of women than they can handle. On the other hand, it would undermine patient’s choice of facilities.

### Study limitations

The findings of this study may have been influenced by study limitations. First, during the study there was no information on quality of care in the LTFP methods of the voucher management program, therefore, this would be a gap that other future researchers willing to evaluate the OBA project would consider to help add information to the literature. Secondly, the study utilized generalized secondary data without considering the inimitable findings for every year of roll out of the voucher scheme hence biased. There may have been unique challenges faced every year and; therefore, future researchers may consider conducting a time series analysis to evaluate the trends of LTFP methods. Thirdly, using the year 2008 as the baseline for the difference in difference calculation may have exuded some biases; however, since the preceding years of 2006 and 2007 in which the voucher scheme were rolled did not have information on non-OBA clients, 2008 provided a relatively good baseline to calculate the difference in difference estimates. There may have been other causes of the difference in access rates between OBA and non-OBA clients which this study did not elucidate; therefore, other researchers may look deeper into that. Nyando and Mwingi were introduced in the Kenya OBA scheme in 2013 which may have brought out some biases and therefore the authors suggests that this data should be used with caution.

## Conclusion

The OBA voucher management scheme is a demand side financing system which provides incentives among service providers to improve on efficiency and quality of health care and shifts purchasing power to consumers (clients) and empowers the patient to choose which facility to visit based on his/her preference or perception of quality of service. The participating service providers (usually a mixture of public, private, FBO and NGO facilities) are made accessible to the voucher holder thereby dismantling financial barriers and stimulating demand for services. The system has provided subsidy using the most cost-effective interventions, promotes public-private partnership and uses competitive approaches to minimise costs and improve the quality of health care services provided. Limitations notwithstanding, this study shows that the OBA voucher scheme in Kenya has been effective in providing LTFP services and improving access of care provided to women of reproductive age. The study strengthens the findings for voucher scheme use as a tool for bridging the gap of unmet needs of family planning in Kenya and could potentially be more effective if rolled out to other counties. However, care needs to be taken so in the spirit of competition within the voucher management system the issues of quality skimping, especially when the facility have taken greater number of women than they can handle, is well sorted.

## Abbreviations

FBOs: Faith Based Organisations
KDHS: Kenya Demographic Health Survey
LTFP: Long-Term Family Planning Methods
MDGs: Millennium Development Goals
NGOs: Non-Governmental Organisations
OBA: Output Based Approach
SGBV: Sexual Gender Based Violence
SMH: Safe Motherhood
